# Hemophagocytic Lymphohistiocytosis Secondary to Miliary Tuberculosis in a Resource-Limited Setting: A Case Report

**DOI:** 10.7759/cureus.73733

**Published:** 2024-11-15

**Authors:** Arthur E McKinnon

**Affiliations:** 1 Internal Medicine, Rob Ferreira Hospital, Nelspruit, ZAF

**Keywords:** advanced hiv disease, hemophagocytic lymphohistiocytosis, human immunodeficiency virus, hyperferritinemia, tuberculosis

## Abstract

Hemophagocytic lymphohistiocytosis is a rare life-threatening condition, with a high mortality rate, characterized by a dysfunctional immune response resulting in multi-organ damage. The secondary or sporadic form of the disease can be triggered by a multitude of infections, malignancies, and autoimmune disorders. Tuberculosis is commonly involved as a trigger for hemophagocytic lymphohistiocytosis.

In a rare case, a 52-year-old gentleman with advanced human immunodeficiency virus (HIV) and disseminated tuberculosis (TB) developed secondary hemophagocytic lymphohistiocytosis. This patient was successfully treated with the 1994 hemophagocytic lymphohistiocytosis treatment protocol at a hospital in a rural province in South Africa. TB, a treatable condition, remains the most lethal disease in South Africa. This case highlights another severe complication of TB. Despite the widespread rollout of antiretroviral treatment, HIV still remains South Africa's largest epidemic. This case emphasizes the need for physicians, treating people living with HIV infection and TB, to have a high index of suspicion in the appropriate clinical situation for hemophagocytic lymphohistiocytosis. Improved outcomes are possible for these cases when diagnosed and treated timely and correctly.

## Introduction

Hemophagocytic lymphohistiocytosis (HLH) is a rare life-threatening condition epitomized by dysfunctional immune regulation involving extreme and dogged activation of macrophages, cytotoxic T lymphocytes, and natural killer cells (NKC) as well as organ infiltration by lymphocytes and histiocytes and resultant organ damage due to excessive inflammatory cytokine production [1,2]. HLH is clinically characterized by recurrent fever, liver dysfunction, cytopenias, organomegaly, hemophagocytosis in the reticuloendothelial system, hypofibrinogenemia, hypertriglyceridemia, and hyperferritinemia. HLH is classified as familial or primary, more common in the pediatric population, and sporadic or secondary, affecting all age groups. Conditions such as infections, malignancy, and autoimmune diseases can trigger HLH [3]. The most common trigger for HLH is infection, with *Mycobacterium tuberculosis* (MTB) being the most common bacterial pathogen implicated. MTB is involved in up to 25% of HLH initiated by infection [1,4]. The incidence of HLH in people living with human immunodeficiency virus (HIV)/acquired immunodeficiency syndrome (AIDS) (PLHA) is not well known [1,3]. The HIV epidemic is still South Africa's (SA's) largest epidemic, with a recent report detailing that approximately 7.8 million people are infected in SA [5]. Furthermore, SA has the highest percentage of patients with advanced HIV disease (AHD), specified as a CD4 of less than 200 cells/mm^3^ or a World Health Organization (WHO) stage III or IV occurrence in adolescents or adults, in sub-Saharan Africa (SSA) [6]. Additionally, SA continues to have a high prevalence and incidence of tuberculosis (TB). Furthermore, TB contributes greatly to SA's mortality and remains to be the leading cause of death in SA [7]. There is evidence to suggest that PLHA are at increased risk of developing HLH [3,4]. HLH is a challenging disease to diagnose and treat, and this case aims to raise awareness among physicians of this life-threatening condition in PLHA.

## Case presentation

A 52-year-old gentleman with AHD and a CD4 count of 113 cells/µL, WHO stage IV, not on antiretroviral treatment (ART) diagnosed with miliary TB on an intensive phase of anti-tuberculous treatment (ATT) presented to our hospital. The patient was diagnosed at his local clinic, by sputum TB GeneXpert, with drug-sensitive TB. Our hospital is in a rural province of SA and forms part of a network of hospitals providing care to the majority of the patients in the province. The patient's main complaint included generalized body weakness, shortness of breath, and recurring fever for the past three days. Additionally, he reported that he had lost more than 10 kg over the last six months. He reported that he is not improving on the ATT. He had been aware of his HIV diagnosis for seven years but had not sought treatment due to fear of stigmatization. He had no history of other medical conditions or relevant social or occupational history.

Physical examination revealed the following: blood pressure 92/65 mmHg, pulse rate 116 bpm, respiratory rate 26 bpm, pulse oximetry 90% on room air and 100% on 2 L of nasal prong oxygen therapy, and fever of 38.9°C. Furthermore, he had generalized lymphadenopathy involving the axillary, posterior, and anterior cervical as well as the inguinal chains and pallor of the conjunctivae. The largest nodes were approximately 3×2 cm. Diffuse fine pan inspiratory crackles were noted on chest auscultation. Prominent hepatomegaly with a liver span of 21 cm and a splenomegaly extending 8 cm below the costal margin were palpated. Dyschromonychia (DCO) was noted on examination of the hands and nails. The former is a change in nail and nailbed pigmentation typically with loss of the lunula and with a blue-grey hue extending from the base of the nail commonly involving less than half of the nail. This sign is associated with HIV infection, especially AHD. This sign is specifically used in the HIV population and should not be used to describe nail dyschromia/chromonychia/nail dystrophy of other causes such as fungal infections, primary nail or skin conditions, nutritional deficiencies, or other miscellaneous causes [8]. Our leading differential diagnosis at this stage included (1) disseminated TB, (2) a lymphoproliferative disorder, and (3) a non-tuberculous mycobacterial infection.

**Figure 1:**
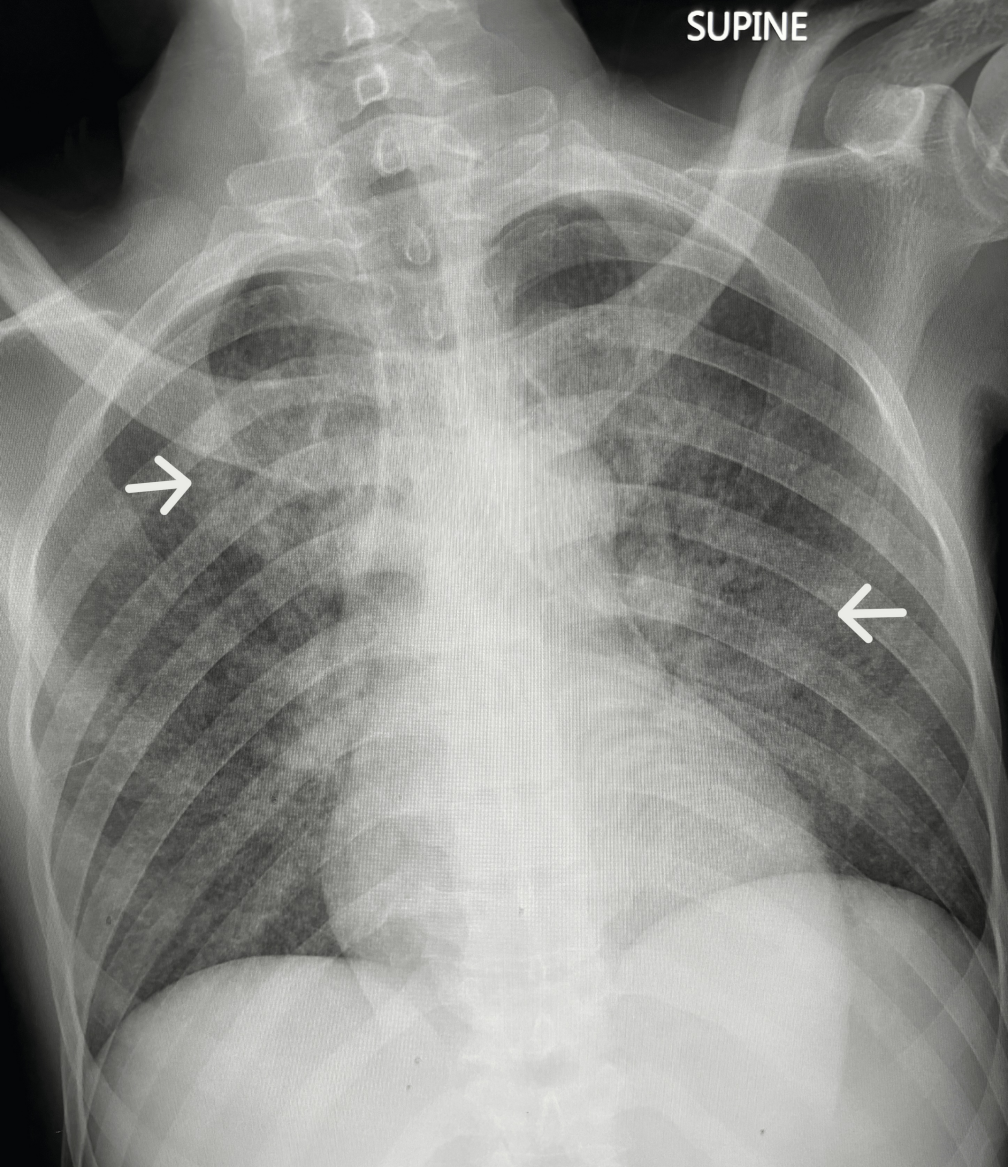
Anteroposterior chest roentgenogram demonstrating reticulonodular infiltrates (white arrows)

**Figure 2:**
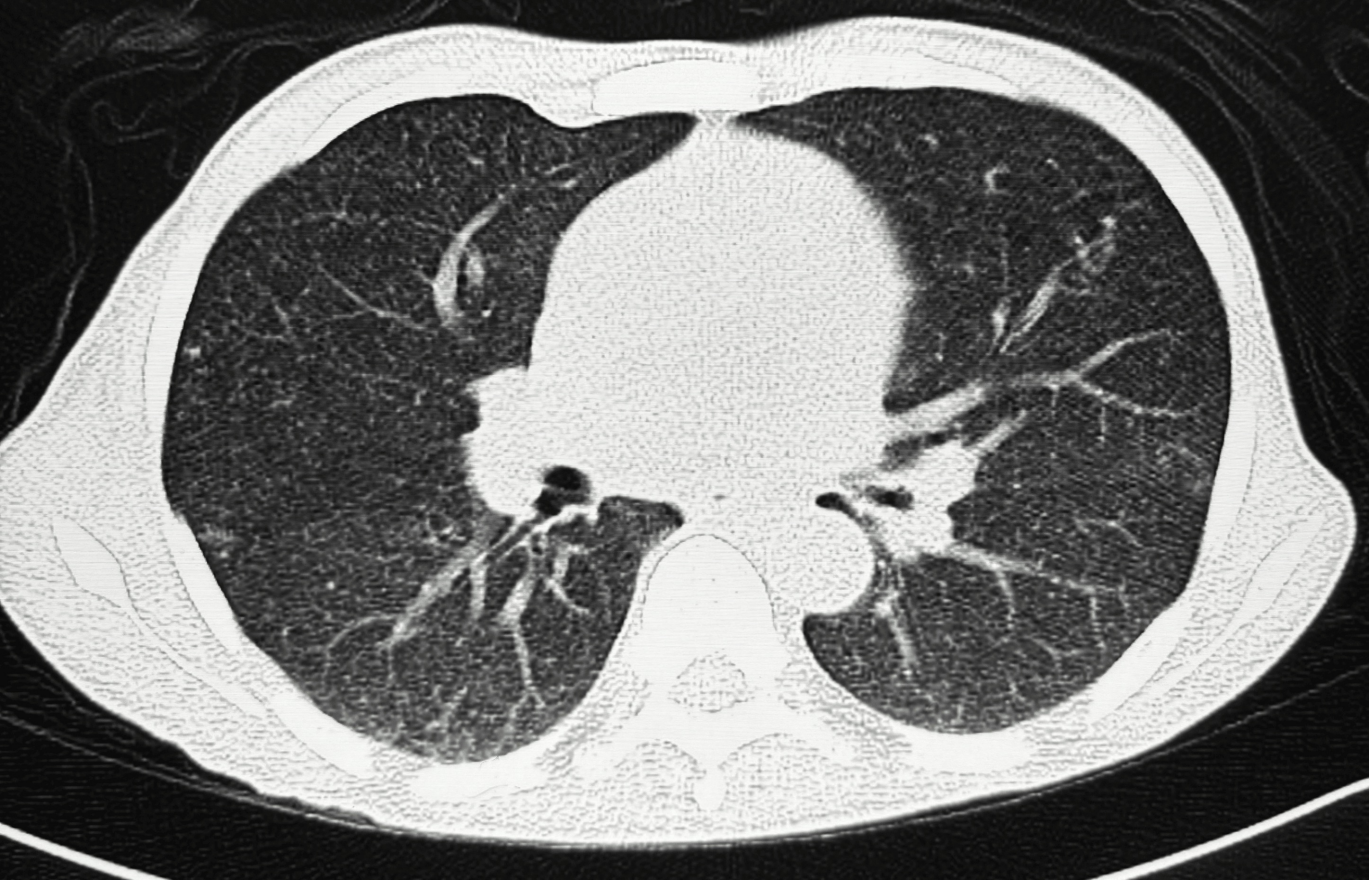
Axial CT of the chest demonstrating pulmonary nodules in a miliary pattern CT: computed tomography

**Figure 3:**
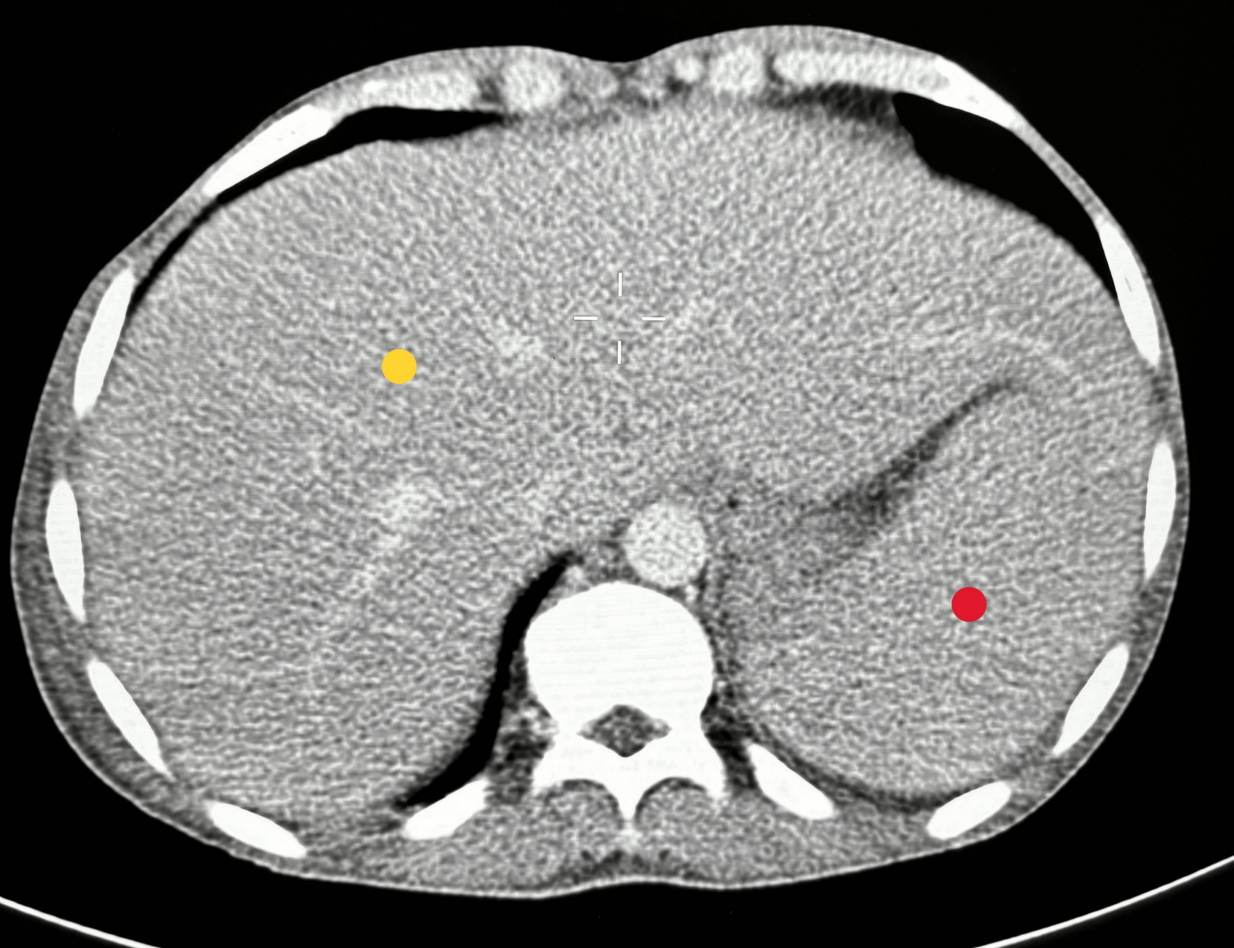
Axial CT of the abdomen demonstrating hepatosplenomegaly: hepatomegaly indicated by a yellow dot and splenomegaly indicated by a red dot CT: computed tomography

The radiological investigation was significant for a miliary pattern on the chest roentgenogram (Figure 1). Computed tomography of the chest and abdomen revealed a millet seed pattern of lung infiltration (Figure 2) as well as a hepatosplenomegaly (Figure 3).

On biochemical investigation (Table 1), he had pancytopenia, with a white cell count of 2.77×109/L, a neutrophil absolute count of 2.28×109/L, hemoglobin (Hb) of 4.9 g/dL, and a platelet count of 67×109/L. He had an iron transfer block, with ferritin of >1500 ng/mL, iron of 12 µmol/L, transferrin of 1.13 g/L, and % saturation of 42%. Additionally, he had elevated serum liver enzymes, with alanine aminotransferase of 134 U/L, aspartate aminotransferase of 416 U/L, alkaline phosphatase of 614 U/L, gamma-glutamyl transferase of 374 U/L, and elevated total bilirubin of 53 µmol/L with a direct bilirubin of 32 µmol/L. The C-reactive protein was 108 mg/L and erythrocyte sedimentation rate (ESR) >120 mm/hr. The patient was hypercalcemic, with albumin-adjusted calcium of 2.77 mmol/L, and his serum magnesium and phosphate were within normal limits. Moreover, he had hypertriglyceridemia with triglycerides of 5.18 mmol/L. He had a low absolute CD4 lymphocyte count of 113 cells/µL. Furthermore, he had acute renal failure, with a creatinine of 184 µmol/L, a blood urea nitrogen (BUN) of 13.1 mmol/L, a BUN/creatinine ratio of 17.74, and an estimated glomerular filtration rate (eGFR) of 36 mL/min. He had a positive lateral flow urine lipoarabinomannan assay test. The former is a point-of-care test for TB that detects the presence of mycobacterial lipoarabinomannan antigen in urine. This antigen is present in active TB disease. Hepatitis B and C investigations, Epstein-Barr virus serology test, cryptococcal antigen test, and prostate-specific antigen test were normal. 

**Table 1:** Laboratory investigations ALT: alanine aminotransferase; ALP: alkaline phosphatase; AST: aspartate aminotransferase; BUN: blood urea nitrogen; CrAg: cryptococcal antigen; EBV: Epstein-Barr virus; eGFR: estimated glomerular filtration rate; ESR: erythrocyte sedimentation rate; GGT: gamma-glutamyl transferase; LF-ULAM: lateral flow urine lipoarabinomannan

Analyte	Result	Reference values
White cell count	2.77×10^9^/L	3.92-10.40
Neutrophil absolute count	2.28×10^9^/L	1.50-8.00
Hemoglobin	4.9 g/dL	13.4-17.5
Platelet count	67×10^9^/L	171-388
Ferritin	>1500 ng/mL	30-400
Iron	12 µmol/L	10-30
Transferrin	1.13 g/L	2.2-4
% saturation	42%	20-50
ALT	134 U/L	5-20
AST	416 U/L	0-30
ALP	614 U/L	47-119
GGT	374 U/L	4-24
Total bilirubin	53 µmol/L	29-42
Direct bilirubin	32 µmol/L	0-3
C-reactive protein	108 mg/L	<10
ESR	>120 mm/hr	<20
Albumin-adjusted calcium	2.77 mmol/L	2.2-2.6
Triglycerides	5.18 mmol/L	<1.7
Absolute CD4 lymphocyte count	113 cells/µL	500-1,500
Creatinine	184 µmol/L	64-104
BUN	13.1 mmol/L	2.1-7.1
eGFR	36 mL/min	>60
CrAg test	Negative	
EBV serology	Negative	
LF-ULAM assay	Positive	
Sputum TB GeneXpert	Positive, rifampicin sensitive	

At this stage, our team was concerned that the patient may have developed HLH secondary to miliary TB. A bone marrow aspirate and trephine biopsy were done: overt hemophagocytosis was demonstrated as well as areas of granulomatous inflammation with caseous necrosis. The Ziehl-Neelsen (ZN) stain was negative. The marrow ferritin was >15,000 ng/mL. An excisional lymph node biopsy of one of the right posterior chain nodes showed granulomatous inflammation with caseous necrosis, the ZN stain was negative, and the TB complex polymerase chain reaction was negative. The patient had already been receiving ATT at the time of the biopsies.

The patient was diagnosed with HLH secondary to miliary TB using the HLH-2004 diagnostic criteria (Table 2) [9]. He fulfilled a total of 6/8 criteria. Soluble interleukin-2 (IL-2) receptor (also soluble CD25) and flow cytometry for NKC activity is not performed at our facility and was therefore not used to calculate the patient's score.

**Table 2:** HLH-2004 diagnostic criteria HLH: hemophagocytic lymphohistiocytosis; sCD25: soluble cluster of differentiation 25; IL-2: interleukin 2; NKC: natural killer cell Reference: [9]

The diagnosis of HLH can be established if criterion 1 or 2 is fulfilled
1. A molecular diagnosis consistent with HLH
2. Diagnostic criteria for HLH fulfilled (five of the eight criteria below):
Fever
Splenomegaly
Cytopenias (affecting ≥2 of three lineages in the peripheral blood)
Fasting triglycerides ≥3 mmol/L and/or fibrinogen ≤1.5 g/L
Hemophagocytosis in the bone marrow or spleen or lymph nodes. No evidence of malignancy
Ferritin ≥500 µg/L
sCD25 (i.e., soluble IL-2 receptor) ≥2400 U/mL
Low or no NKC activity (according to local laboratory reference)

Our patient was initially treated with intravenous (IV) fluid therapy at rates of 125 mL/hr. He was placed in an isolation room, and IV broad-spectrum antibiotics along with ATT and Bactrim prophylaxis were started. The patient was transfused with cross-matched red blood cells (RBC) to achieve an Hb of >10 g/dL. A higher Hb transfusion target was used to comply with the hospital chemotherapy guidelines in order to prepare the patient for his etoposide phosphide (ETOP) treatment. Additionally, he was treated with IV dexamethasone. Oxygen therapy was titrated according to the patient's requirements. Once the patient achieved an Hb of >10 g/dL, a neutrophil absolute count of >1.5×109/L, and a platelet count of >100×109/L, he was started on the HLH-94 treatment protocol [9]. ETOP was initially given as 50% of the recommended IV dose, i.e., 75 mg/m^2^, due to the patient's renal failure. Kidney function improved to normal after the third IV dose of ETOP. Thereafter, ETOP was administered at 150 mg/m^2^ IV for the duration of treatment. Dexamethasone was given daily at 10 mg/m^2^ IV for the first two weeks and then tapered over the following weeks according to the HLH-94 treatment protocol. Cyclosporine A was not administered because it was unavailable at the treating facility. RBC and platelets were transfused as needed in order to uphold the patient's fitness for chemotherapy. Over the intensive course of chemotherapy, the patient slowly recovered to pre-morbid functional capability. During his hospital stay, he received routine treatment sessions from the physiotherapy and occupational therapy department staff. The dietetics department was also extensively involved in the overall treatment and care of this patient. In total, he completed eight weeks of therapy, and at that time, it was decided not to offer the patient a continuation chemotherapy plan. ART was started after eight weeks of ATT. The chosen regimen of ART included abacavir 600 mg/lamivudine 150 mg/dolutegravir 50 mg with an additional dolutegravir 50 mg booster due to its interaction with rifampicin. The patient was transitioned to tenofovir 300 mg/lamivudine 300 mg/dolutegravir 50 mg as a fixed dose combination at the HIV clinic after his kidney function returned to normal. Dolutegravir was boosted until he completed his ATT. The patient was then followed up on a monthly basis until he completed a total of nine months of ATT. He was assessed as cured of TB. The patient was hospitalized for a total of 59 days. Currently, his HIV is well controlled, and he follows up at our HIV clinic on a six-monthly basis. His last HIV viral load was lower than the detectable limit with an absolute CD4 cell count of 199 cells/µL.

## Discussion

A reductionist approach is often applied in medicine, the so-called Occam's razor, but in AHD, this approach may have insufficient diagnostic power. PLHA, especially those with AHD, are at risk of multiple infections, their complications, as well as oncological conditions at the same time. This should be kept in mind when treating PLHA [10]. HLH carries a high mortality rate, even with treatment, with survival rates being reported between 40% and 60% [1,4]. Effective and timely treatment has been shown, in case reports from the Free State SA, to decrease mortality from 95% to 35% [11]. There is very little research on HLH in PLHA, reflected in a review of cases reported between 2005 and 2021 by Tabaja et al., identifying only 81 clinical cases in total described in the literature in that timeframe [3]. The former highlights the need for more research in this area. PLHA are at increased risk of developing HLH. This is because HIV also serves as a trigger for HLH and these patients are at increased risk of opportunistic infections that similarly serve as secondary triggers [3,10]. A recent review by Kurver et al. revealed a total of 115 cases of HLH in HIV-negative individuals with confirmed TB [12]. This highlights the need for the treating physician to include HLH in their differential diagnosis in both HIV-negative and PLHA. The diagnosis of HLH in PLHA is challenging and requires a high index of suspicion. HLH, TB, and AHD share numerous clinical and biochemical criteria listed in the diagnostic criteria for HLH. This may complicate the diagnosis of HLH in this population group and delay diagnosis and treatment, as well as lead to underdiagnosis. Previous case studies published reflect on the difficulty of making this diagnosis [3,13,14]. Furthermore, extra-pulmonary TB as a trigger for HLH is probably underreported, and it is advised that bone marrow investigations should be part of the diagnostic workup of all HLH patients to investigate for TB [12]. A lack of awareness among treating physicians as well as delay in diagnosis, even in intensive care settings, can unnecessarily increase the morbidity and mortality of these patients [3,11]. SA's HIV and TB epidemic should prompt physicians to stay alert for complications associated with these conditions that carry high mortality rates and are treatable [5,11,15]. 

## Conclusions

This case demonstrates a rare case of HLH secondary to miliary TB in a patient with AHD. Furthermore, it highlights that improved outcomes are possible for this life-threatening disorder, even in a resource-limited setting, if prompt diagnosis and effective treatment are initiated. Additionally, it shows the complexity of disease presentation in PLHA. This case emphasizes the need for a high index of suspicion among physicians treating PLHA and TB for HLH.
